# Drug resistance profiles of *Mycobacterium tuberculosis* clinical isolates by genotype MTBDRplus line probe assay in Zambia: findings and implications

**DOI:** 10.1093/jacamr/dlae122

**Published:** 2024-07-25

**Authors:** Mundia Hendrix Kangongwe, Winnie Mwanza, Mutende Mwamba, Jonathan Mwenya, John Muzyamba, Judith Mzyece, Amos Hamukale, Emmanuel Tembo, Davy Nsama, Rehab Chimzizi, Angel Mubanga, Bushimbwa Tambatamba, Steward Mudenda, Kennedy Lishimpi

**Affiliations:** Ministry of Health, Chest Diseases Laboratory, Lusaka, Zambia; Institute for Basic and Biomedical Sciences, Levy Mwanawasa Medical University, Lusaka, Zambia; Ministry of Health, National Tuberculosis and Leprosy Programme, Lusaka, Zambia; Public Health, USAID-STAR Project; Ministry of Health, Chest Diseases Laboratory, Lusaka, Zambia; Ministry of Health, Chest Diseases Laboratory, Lusaka, Zambia; Ministry of Health, Chest Diseases Laboratory, Lusaka, Zambia; Ministry of Health, Laboratory and Pathological Services, Lusaka, Zambia; Epidemiology and Surveillance, Zambia National Public Health Institute, Lusaka, Zambia; Ministry of Health, National Tuberculosis and Leprosy Programme, Lusaka, Zambia; Ministry of Health, Laboratory and Pathological Services, Lusaka, Zambia; Ministry of Health, National Tuberculosis and Leprosy Programme, Lusaka, Zambia; Public Health, USAID-STAR Project; Ministry of Health, National Tuberculosis and Leprosy Programme, Lusaka, Zambia; Technical Services, Ministry of Health Headquarters, Lusaka, Zambia; Department of Pharmacy, School of Health Sciences, University of Zambia, Lusaka, Zambia; Technical Services, Ministry of Health Headquarters, Lusaka, Zambia

## Abstract

**Background:**

The emergence of drug resistance is a threat to global tuberculosis (TB) elimination goals. This study investigated the drug resistance profiles of *Mycobacterium tuberculosis* (*M. tuberculosis*) using the Genotype MTBDRplus Line Probe Assay at the National Tuberculosis Reference Laboratory (NTRL) in Zambia.

**Methods:**

A cross-sectional study was conducted between January 2019 and December 2020. GenoType MTBDRplus line probe assay records for patients at the NTRL were reviewed to investigate drug susceptibility profiles of *M. tuberculosis* isolates to rifampicin and isoniazid. Data analysis was done using Stata version 16.1.

**Results:**

Of the 241 patient records reviewed, 77% were for females. Overall, 44% of patients were newly diagnosed with TB, 29% had TB relapse, 10% treatment after failure and 8.3% treatment after loss to follow-up. This study found that 65% of *M. tuberculosis* isolates were susceptible to rifampicin and isoniazid. Consequently, 35% of the isolates were resistant to rifampicin and/or isoniazid and 21.2% were multidrug-resistant (MDR). Treatment after failure [relative risk ratios (RRR) = 6.1, 95% CI: 1.691–22.011] and treatment after loss to follow-up (RRR = 7.115, 95% CI: 1.995–25.378) were significantly associated with MDR-TB. Unknown HIV status was significantly associated with isoniazid mono-resistance (RRR = 5.449, 95% CI: 1.054–28.184).

**Conclusions:**

This study found that 65% of *M. tuberculosis* isolates were susceptible to rifampicin and isoniazid while 35% were resistant. Consequently, a high prevalence of MDR-TB is of public health concern. There is a need to heighten laboratory surveillance and early detection of drug-resistant TB to prevent the associated morbidity and mortality.

## Introduction

Tuberculosis (TB) is a communicable disease that is a major cause of ill health and one of the leading causes of death worldwide.^1,2^ Globally, TB remains an important public health problem, with about 10.6 million people developing TB in 2022.^3^ A total of 1.3 million people died from TB in the year 2022 of which 167 000 had human immunodeficiency virus (HIV) as a comorbid infection.^3^ Until the coronavirus disease 2019 (COVID-19) pandemic, TB was the leading cause of death from a single infectious agent, ranking above HIV/AIDS.^3–6^

The fight to end TB by 2035 as enshrined in the WHO End TB Strategy is threatened by the emergent spread of drug-resistant TB (DR-TB), in particular, multidrug- or rifampicin-resistant TB (MDR/RR-TB).^7–9^ MDR-TB is caused by *Mycobacterium tuberculosis* complex (MTBC) bacteria with resistance to rifampicin (RIF) and to isoniazid (INH).^8,10^ In other words, MDR-TB is a form of TB caused by bacteria that do not respond to isoniazid and rifampicin, the two most effective first-line TB drugs.^3,8^ The resistance of MTBC to anti-tuberculosis drugs is a global public health problem.^11–13^ In 2018, the WHO reported about half a million new cases of RIF mono-resistant TB (RR-TB), of which 78% had MDR-TB.^14^ In addition, an estimated 830 000 people had TB disease due to INH mono-resistant strains of MTBC.^14^ Drug-resistant TB occurs through intrinsic and extrinsic mechanisms.^8,10,15^ Four years later in 2022, approximately 410 000 people suffered from MDR/RR-TB.^3^ Moreover, drug-resistant TB is worsened by the inappropriate use of TB medicines including through incorrect prescription by healthcare workers, poor quality drugs, non-adherence to treatment and when patients stop treatment prematurely.^3,16–19^ Therefore, there is a need to promote the rational prescribing of TB drugs and improve patient adherence to their TB medicines.^20,21^

To meet the End TB Strategy targets, WHO recommended the use of molecular rapid TB diagnostics (mWRDs) on all individuals with signs or symptoms of TB, and universal access to drug susceptibility testing (DST) for at least RIF on all bacteriologically confirmed TB patients, and all patients with RR-TB should receive DST for at least fluoroquinolones (FQs).^14,22^ To scale up early detection and commence patients on treatment, testing platforms with high diagnostic accuracy are key to meeting TB elimination goals. Moreover, rapid identification of drug-resistant TB reduces the risk of patient mismanagement, the amplification of drug resistance and ongoing transmission.^23^

Tuberculosis treatment outcomes are poor in low- and-medium-income countries (LMICs) due to low education, low income, under-nutrition, high alcohol intake, rural residence, drug-shortages, stigma, long travel times, a lack of social protection and high HIV burden.^24,25^ Consequently, the burden of TB is high in LMICs that often bear the highest morbidity and mortality rates due to this disease.^26,27^ Other regions with the highest TB morbidity and mortality rates include Southeast Asia, West Pacific regions and African regions.^27,28^ Additionally, the impact of drug-resistant TB is highest in LMICs that have also reported higher morbidity and mortality rates associated with MDR-TB.^29,30^

Like in other LMICs, TB continues to be a major public health problem in Zambia.^31,32^ The country is situated in the sub-Saharan African (SSA) region and is among the countries with the highest burden of TB in Africa.^33^ With an estimated incidence rate of 319/100 000 population, Zambia ranks just above Botswana and below South Africa in the region and is classified among the 30 countries with the highest per capita burden of TB in the world.^34^ The main driver of the TB epidemic in Zambia is HIV.^32,35^ This is evident from the 2022 Global TB report that reported that Zambia is among the countries with a high burden of TB, HIV-associated TB, and MDR/RR-TB.^5^

In Zambia, the burden of MDR-TB has been reported to be on the increase.^5^ For instance, a study that reviewed TB national data observed a 4-fold increasing trend in MDR-TB cases being detected by the National Tuberculosis & Leprosy Program (NTLP) between 2000 and 2011.^36^ The proportions of MDR-TB have risen from 0.3% among new cases and 8.1% among previously treated cases in 2014 to 2.8% in new cases and 18% in previously treated cases in 2019.^37^ Most recently, Zambia has been reclassified as one of the high MDR/RR-TB burden countries.^34^ This study investigated the drug resistance profiles of *M. tuberculosis* using the Genotype MTBDRplus Line Probe Assay at the National Tuberculosis Reference Laboratory (NTRL) in Lusaka, Zambia.

## Materials and methods

### Study design, setting and population

**Figure 1.**
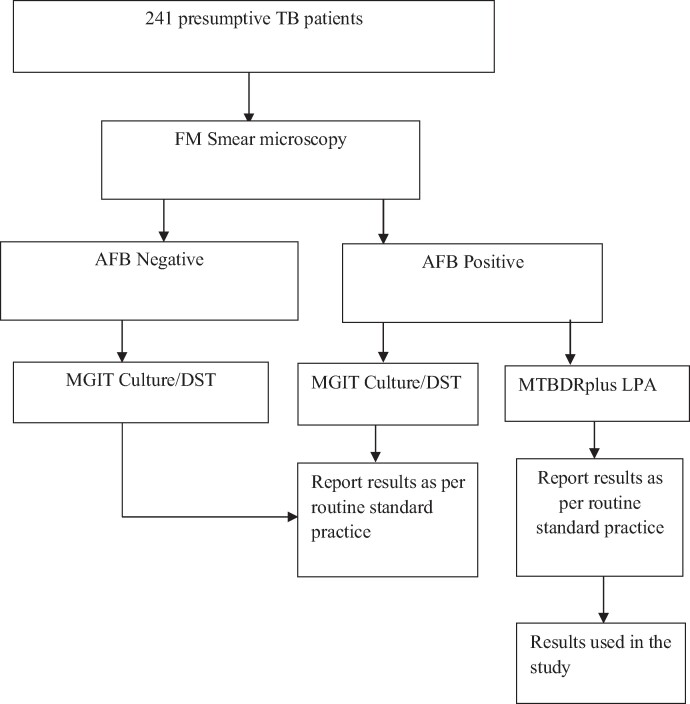
Sample process flow for *M. tuberculosis* isolates used in the study.

This was a laboratory-based cross-sectional study that utilized secondary data at the National Tuberculosis Reference Laboratory (NTRL) and was conducted between June 2021 and December 2021. For this study, we included all successful GenoType MTBDRplus Line Probe Assay results obtained between January 2019 and December 2020. The NTRL is accredited to ISO 15189:2012 by the Southern African Development Community Accreditation Services (SADCAS). It is the largest referral laboratory in Zambia and serves as the supervising laboratory for all 492 TB laboratories in the country’s TB laboratory diagnostic network. The NTRL receives referral samples from confirmed and presumptive DR-TB patients across five provinces (Southern, Eastern, Central, Muchinga, and Northern Province) of Zambia for treatment monitoring and DST. Tests performed at the NTRL include liquid and solid cultures, and phenotypic and Genotypic DST for both first and second line drugs. For all patients whose results were included, demographic information and treatment history were reviewed and included for analysis. The procedure used in this study is outlined in Figure 1.

### Decontamination of sputum

Before extraction of genomic DNA for line probe assay, sputum samples were initially decontaminated using the N-acetyl L-cysteine and sodium hydroxide (NALC-NaOH) procedure for sputum decontamination following standard operating procedure at the NTRL (CDL-TB-TECH-003). An equal volume of NALC-NaOH solution was added to each specimen in a 50 mL conical centrifuge, followed by tightly tightening the screw cap. The mixture was mixed by vortexing for at least 20 s inside a certified class II biosafety cabinet ensuring that the content touched the cap for effective decontamination. The centrifuge tubes were left to stand for 20 min at room temperature after which sterile phosphate buffer was added to the 50 mL mark to neutralize the NALC-NaOH. With the screw cap tightened the tube was inverted several times for thorough mixing. The tubes were centrifuged at 3000 × g for 20 min in aerosol-free sealed centrifuge buckets. The mixture was carefully decanted into a splashproof container containing a mycobactericidal disinfectant such as 5% phenol, leaving a pellet at the bottom of the tube. The pellet was subsequently re-suspended in 2 mL of phosphate buffer and then used for smear microscopy, extraction of genomic DNA and inoculation into MGIT culture tubes.

### GenoType MTBDRplus line probe assay

The GenoType MTBDRplus assay (Hain Lifescience, GmbH, Nehren, Germany) is based on DNA-strip technology, which permits the detection of *M. tuberculosis* complex and susceptibility to RIF and INH.^38^

The MTBDRplus line probe assay involved three procedures that included DNA extraction, amplification, and hybridization. The procedures are outlined below and have been reported in similar studies.^39–42^

### Extraction and amplification of DNA

Extraction of genomic DNA from decontaminated samples was done using commercially prepared GenoLyse reagents A-LYS and A-NB. The procedure was done as outlined in the standard operating procedure (SOP).^38^

Preparation of the master mix was done by adding 10 µL AM-A [amplification Mix A] and 35 µL AM-B [amplification mix B] in a clean room free from DNA contamination. The entire procedure was done as outlined in the SOP.^38^

### Hybridization of DNA amplicons

After completion of the amplification process, the amplicons were detected following the procedure for hybridization from the manufacturer’s kit insert adopted and customized at the NTRL according to standard operating procedure number CDL-TB-TECH-011.

Interpretation of drug resistance profiles of MTBC strains was done by visualizing patterns of bands representing lines of immobilized probes bound (or hybridized) to MTBC amplicons on the DNA strips. This was done with the aid of a manufacturer-provided interpretation card.^38^

### Study measures

This study addressed drug-resistant TB including MDR-TB, mono-resistant TB, relapse, treatment after failure, and treatment after loss to follow-up. In this study, MDR-TB was defined as resistance to both isoniazid and rifampicin, in combination with other drugs or not.^43^ Further, mono-resistant TB is TB resistant to one first-line anti-TB drug only. Furthermore, isoniazid resistant TB is TB resistance to isoniazid while rifampicin-resistant TB is TB resistant to rifampicin. In this study, according to the Zambia TB and leprosy guidelines, relapsed TB patients were classified as patients previously treated for TB and declared cured, or who have completed their most recent course of treatment and are diagnosed with an episode of TB that is either a true relapse or a new episode caused by reinfection.^43^ Treatment after failure patients were classified as patients previously treated for TB and their most recently completed course of treatment failed. Treatment after loss to follow-up patients were classified as patients previously treated for TB and declared ‘lost to follow-up’ at the end of their most recent course of treatment.^43^

### Data analysis

Data were extracted from the laboratory information system (LIS) and analysed using Stata version 16.1. We used descriptive statistics to summarize the sociodemographic characteristics of patients and depict the sociodemographic variables and proportions of drug-resistant TB. Relative risk ratios (RRR) at 95% confidence intervals (95% CI) were calculated to examine the association between the risk factors and drug resistance. Bivariate analysis was used to evaluate the independent association between TB drug resistance, treatment history and HIV status. We considered all the associations with *P* values less than 0.05 statistically significant.

### Ethical approval

This study was conducted as a routine activity from the Chest Diseases Laboratory and was approved by the Zambia National Health Research Authority (NHRA) with approval number 1203/19/05/2024.

## Results

A total of 241 MTBC isolates from 241 patients’ records had successful first-line Genotype MTBDRplus test results in the period under review and were included in this evaluation. The median age for study participants was 36 years (IQR 30–45). Females represented 77% (185/241) of patients, 31% were HIV positive, 44% were newly diagnosed while 29%, 10% and 8.3% were relapse cases, treatment after failure and treatment after loss to follow, respectively (Table 1).

**Table 1.** Baseline sociodemographic characteristics of study participants (*N* = 241)

Category	No (%)
Sex	
Male	55 (22.8)
Female	185 (76.8)
Unknown	1 (0.4)
Treatment history	
New	106 (44)
Relapse	70 (29)
Treatment after failure	24 (10)
Treatment after loss to follow-up	20 (8.3)
Unknown	21 (8.7)
HIV status	
Negative	120 (49.8)
Positive	74 (30.7)
Unknown	47 (19.5)

Overall, the drug susceptibility pattern showed that 65% (157/241) of isolates were sensitive to RIF and INH while 35% (84/241) showed resistance to INH and/or RIF. Consequently, 21.2% of the isolates were MDR, 7.1% were RIF mono-resistant and 6.6% were INH mono-resistant (Table 2). Of the 51 MDR cases, 14 (27.5%) were new TB cases, while 32 (62.7%) were retreatment cases including 16 (31%) relapse cases, 8 (16%) cases of treatment after failure and 8 (16%) cases of treatment after loss to follow-up (Table 2).

**Table 2.** Rifampicin and isoniazid resistance patterns versus patient treatment history

Patient characteristic	Susceptible to INH and RIF	Resistant to INH and RIF	RIF mono-resistance	INH mono-resistance
*N* = 241	*n* = 157 (65%)	*n* = 51 (21.2%)	*n* = 17 (7.1%)	*n* = 16 (6.6%)
New	76 (48%)	14 (28%)	7 (41%)	9 (56%)
Relapse	41 (26%)	16 (31%)	8 (47%)	5 (31%)
Treatment after failure	15 (10%)	8 (16%)	0.0	1 (6%)
Treatment after loss to follow-up	10 (6%)	8 (16%)	2 (12%)	0
Unknown	15 (10%)	5 (10%)	0	1 (6%)

Table 3 shows the relative risk ratios for specific drug resistance profiles of *M. tuberculosis*. The risk of having INH mono-resistance was 5.45 times for patients with unknown HIV status compared to those who were HIV-negative. Additionally, the risk of having MDR-TB among patients with treatment after failure was 6.1 times compared to the new cases. Furthermore, the risks of having MDR-TB among patients with treatment after loss to follow-up were 7.12 times compared to the new cases (Table 3).

**Table 3.** Patient characteristics and their association with rifampicin and isoniazid resistance

Characteristic	Isoniazid mono-resistance			Multidrug-resistance TB		
	RRR	95% CI	*P* value	RRR	95% CI	*P* value
HIV status						
Negative	1	—	—	1	—	—
Positive	0.67	0.114–3.894	0.653	0.65	0.269–1.607	0.358
Unknown	5.45	1.054–28.184	**0**.**043**	0.78	0.202–2.981	0.713
Treatment history						
New	1	—	—	1	—	—
Relapse	0.71	0.156–3.229	0.658	2.65	0.959–7.315	0.06
Treatment after failure	1.26	0.128–12.465	0.842	6.1	1.691–22.011	**0**.**006**
Treatment after LTFU	0	—	0.995	7.12	1.995–25.378	**0**.**002**

The bold values indicate statistical significance.

## Discussion

Our study investigated the drug resistance profiles of *M. tuberculosis* using the Genotype MTBDRplus Line Probe Assay at the NTRL, Zambia for all samples processed between January 2019 and December 2020.

The present study found that 65% of the *M. tuberculosis* isolates were susceptible to INH and RIF. Our findings corroborate with those reported in Ethiopia where 66% of the isolates were susceptible to the first-line anti-TB drugs.^44^ The current study revealed an overall drug resistance of 35% to rifampicin and or isoniazid and the burden of MDR-TB was found to be 21.2%. Additionally, the proportions of MDR-TB in newly diagnosed and previously treated cases in our study were 28% and 31%, respectively. These findings show an increased trend in new MDR-TB cases in our study (28%) compared to 2.8% in 2019, while a lower prevalence of 15.4% among retreatment cases was reported in our study, compared to 18% in 2019.^37^ In keeping with this observation, a more recent study on drug-resistant TB in the Northern region of Zambia reported a much higher prevalence (53%) of MDR-TB in both new and previously treated TB patients compared to our findings.^45^ The high prevalence of MDR-TB in Zambia is a public health concern and requires urgent attention to counteract this problem and the contributing factors. In China, the prevalence of MDR-TB was reported to be 34.85%.^46^ A similar study in Ethiopia found a prevalence of MDR-TB to be 2% in the new cases and 15% in previously treated TB patients.^47^ Our study, however, reported a 14-fold higher proportion of MDR-TB in new TB cases than what was reported in a systematic review and meta-analysis of multidrug-resistant tuberculosis in Ethiopian settings.^47^ Further, the prevalence of MDR-TB reported in our study is greater than the 11.6% reported in a global systematic review and meta-analysis,^48^ 5.4% reported in India,^49^ and 5.4% reported in Botswana,^50^ receptively. These findings demonstrate the increasing resistance of *M. tuberculosis* to anti-TB drugs that requires urgent attention.

The majority of isolates in our study came from female patients. However, MDR-TB was higher in the male folk than their female counterparts. This finding is consistent with some studies conducted in Zambia^45^ and other parts of the globe^51,52^ where most males had MDR-TB compared to females. On the contrary, a South African study reported higher rates of DR-TB, including MDR and XDR-TB in women than in men.^53^ Previous evidence has demonstrated that the risks of developing MDR-TB among patients include living in a rural area, living in houses with more than six people, history of previous TB treatment, history of incomplete TB treatment, non-adherence to treatment, history of cigarette smoking and direct contact with known TB patients.^54–58^

Our study revealed that patients with treatment after failure and treatment after loss to follow-up were at a high risk of developing MDR-TB. This finding is consistent with reports from other studies conducted earlier.^59,60^ Patients with a history of TB treatment failure in our study were at a higher risk of developing MDR-TB. The finding is in tandem with other studies conducted in Ethiopia and a systematic review and meta-analysis involving 28 studies across Europe, Asia and America.^47,60–62^

Our study found that RIF mono-resistance was common among TB patients, especially those who had new cases, relapse and treatment after loss to follow-up with a prevalence of 7.1% recorded. Our finding is lower than the 32.3% reported in an earlier study conducted in the Northern region of Zambia.^45^ Additionally, our study found lower RIF mono-resistant TB compared to a global systematic review and meta-analysis that found a prevalence of RIF mono-resistance TB at 9.4%.^48^ Other studies have reported RIF mono-resistant TB higher than what was found in the present study.^44,53^ Our study and similar studies indicate the need to sensitize patients on the importance of adhering to TB medicines and not dropping out of treatment.^63,64^

Overall, INH mono-resistance in our study population was reported to be 6.6%. This finding represents an upward trend to that reported in an earlier study in Zambia that reported an INH mono-resistance of 3.4%.^45^ However, the prevalence of INH mono-resistance reported in our study was lower compared to the one reported in a global study.^48^ Additionally, many other studies have shown an increase in the prevalence of *M. tuberculosis* resistant to INH.^48,65–68^ In our study, *M. tuberculosis* isolated from patients of unknown HIV status was significantly associated with INH mono-resistance. Additionally, INH mono-resistance was higher in new (9/16; 56%) compared to retreatment cases (6/16; 37.5%). INH mono-resistance is a more common form of drug-resistant TB than RIF mono-resistance and this form of resistance has been associated with positive HIV status, poor treatment outcomes and progression to MDR-TB.^69,70^

Considering that Zambia was recently reclassified by WHO as one of the high MDR/RR-TB burdened countries and coupled with the use of GeneXpert as a screening tool for DR-TB, patients with mono-resistance to INH may inevitably be missed. According to the existing National Tuberculosis & Leprosy Program consolidated guidelines, only patients with RR-TB detected by Xpert and those at high risk for DR-TB are recommended for a full investigation of drug resistance by molecular (e.g. line probe assay) and culture-based (phenotypic) DST. Therefore, treatment of patients with a missed diagnosis of INH resistance (Xpert MTB-Rif ultra) with standard first-line anti-TB drugs is more or less equated to RIF mono-therapy during the continuation phase of treatment. This may subsequently promote the development of drug resistance. Therefore, universal access to DST and, or incorporation of molecular rapid diagnostic platforms with the capacity to simultaneously detect MTB, RIF and INH resistance, is key to identifying and commencing such patients on appropriate therapy to ensure favourable treatment outcomes and curtail development and transmission of resistance. The above findings on drug-resistant TB require the development and implementation of strategies to address this problem and reduce the associated morbidity and mortality.^2,9,22,71–74^ Therefore, countries must ensure they implement several solutions to address TB including developing new diagnostic tests, increasing investments to accelerate the development of the potential new TB vaccines in the pipeline, increasing TB awareness, promoting adherence to anti-TB drugs, implementing new immune strategies for better vaccine design, develop good quality anti-TB drugs, new therapies, implementation of prevention through screening using standardized old and new diagnostic tests for TB infection detection.^75–78^

We are aware that our study had limitations. Our study was a laboratory-based cross-sectional study of patient data in our LIS. The small sample size and lack of clinical outcomes could inevitably impact the generalization of the study findings. However, the findings of this study demonstrate the need to sensitize the Zambian population to seek medical help when they have prolonged respiratory diseases. Subsequently, our findings indicate the need to heighten TB surveillance programmes to assist with early detection and improve treatment outcomes.

### Conclusion

This study revealed that 65% of *M. tuberculosis* isolates were susceptible to rifampicin and isoniazid while 35% were resistant to these first-line drugs. The evidence of MDR-TB reported in our study is of public health concern that requires urgent attention. Therefore, there is a need for heightened laboratory surveillance and early detection of any form of drug resistance coupled with full implementation of universal access to DST for all bacteriologically confirmed TB cases. These strategies are key to addressing drug-resistant TB and are useful for evaluating the success of TB programmes towards meeting TB elimination goals. Finally, addressing drug-resistant TB would help reduce the morbidity and mortality associated with this infectious disease.
